# Supplementary material for: Picosecond pulse-shaping for strong three-dimensional field-free alignment of generic asymmetric-top molecules
Source: Nat Commun. 2022 Mar 17;13:1431. doi: 10.1038/s41467-022-28951-z (PMC8931173; doi:10.1038/s41467-022-28951-z)
Supplement: Supplementary file 1 — Supplementary Information [file 41467_2022_28951_MOESM1_ESM.pdf]

Supplementary Information: Picosecond pulse-shaping for strong three-dimensional field-free alignment of generic asymmetric-top molecules

Terry Mullins,[1] Evangelos T. Karamatskos,[1,2] Joss Wiese,[1,3,4] Jolijn Onvlee,[1,4]
Arnaud Rouzée,[5] Andrey Yachmenev,[1] Sebastian Trippel,[1,4] and Jochen Küpper[1,2,3,4]

[1]*Center for Free-Electron Laser Science, Deutsches Elektronen-Synchrotron DESY, Notkestraße 85, 22607 Hamburg, Germany*
[2]*Department of Physics, Universität Hamburg, Luruper Chaussee 149, 22761 Hamburg, Germany*
[3]*Department of Chemistry, Universität Hamburg, Martin-Luther-King-Platz 6, 20146 Hamburg, Germany*
[4]*Center for Ultrafast Imaging, Universität of Hamburg, Luruper Chaussee 149, 22761 Hamburg, Germany*
[5]*Max Born Institute, Max-Born-Straße 2a, 12489 Berlin, Germany*
(Dated: 2022-02-10)

Email: jochen.kuepper@cfel.de
website: `https://www.controlled-molecule-imaging.org`

SUPPLEMENTARY METHODS: EXPERIMENTAL DETAILS

The peak intensity was determined by combining measurements of the pulse energy, the temporal profile, and the spatial beam profile. The pulse energy was determined by measuring the average power (Coherent PM30 power meter) and dividing by the repetition rate of 1 kHz. The temporal profile of the alignment pulse was determined experimentally by measuring a cross-correlation between the alignment pulse and the Coulomb explosion pulse. Finally, the spatial beam profile was measured on a beam profiler (Ophir Photonics, Spiricon SP620U). We note that the pulse shaper affected both the temporal profile and the transversal spatial distribution of the beam, leading to the so-called space-time coupling. As a result, the temporal profile was not homogeneous spatially and the maximum degree of molecular alignment was achieved experimentally for a probe laser beam that was spatially offset with respect to the alignment laser beam. To take this effect into account, the spectrum of the alignment laser pulse was filtered using a 1 nm band-pass filter centered at a wavelength of 815 nm. This section of the spectrum was chosen as it provided the highest contribution to the intense peak observed in the temporal intensity profile of the alignment laser pulse. We note that the peak intensity of the band-passed alignment laser pulse coincided with the position of the probe laser beam that provided an optimal molecular alignment. The position of this peak was used, in conjunction with the integrated spatial beam profile of the alignment laser, i. e., including all wavelengths, to scale the measured integrated energy, which was only measurable for all combined wavelengths. The scaling factor used, I_{sc}, was defined as the ratio between the intensity measured at the position of the probe laser where maximum alignment was achieved and the peak intensity of the laser beam obtained without filtering. The peak intensity of the alignment laser pulse that is shown in Fig. 2 of the main manuscript was then obtained using the following expression:

$$I_0 = E\, I_{\text{sc}} / \left(\int\!\!\int I_s(x,y)dxdy \int I_t(t)dt \right) \tag{1}$$

with the measured pulse energy E, the normalized spatial intensity profile $I_s(x,y)$ obtained from the beam profiler measurement, and the normalized temporal intensity profile $I_t(t)$ retrieved from the cross-correlation measurement.

The statistical and systematic error of the peak intensity was estimated around ∼7 % and ∼10 %, respectively. We note that the degree of alignment was not significantly changing when the peak intensity was varied by ±10 %, in agreement with our error estimates. This was also confirmed by simulations carried out for different peak intensities.

SUPPLEMENTARY NOTE 1: ION-MOMENTUM DISTRIBUTIONS FROM STRONG-FIELD IONIZATION OF ALIGNED INDOLE

Ion-momentum distributions for H^+, C^{2+} and CH_xN^+(x=0,1,2) fragments recorded at a time delay of $t = 3.3$ ps, i. e., at the highest degree of field-free alignment, are shown in Figure 1 of the main article. In addition, C^+, C_2^+, $C_3H_x^+$, and $C_4H_x^+$ ion-momentum distributions were also recorded in the same experimental conditions and are displayed in Supplementary Figure 1. For $C_3H_x^+$ and $C_4H_x^+$ fragments, x corresponds to fragments with different number of hydrogens whose masses could not be resolved by the high-voltage gating of the detector. In the top row, ion-momentum distributions are shown with the major polarization axis of the alignment laser being parallel and the minor polarization axis being perpendicular to the detector plane ($\alpha = 0°$ in main paper), whereas in the bottom row the major polarization axis is perpendicular and the minor polarization axis is parallel to the detector plane ($\alpha = 90°$ in the main manuscript).

SUPPLEMENTARY NOTE 2: TOMOGRAPHIC RECONSTRUCTION OF THE H^+ 3D MOMENTUM DISTRIBUTION

The 3D momentum distribution of H^+ obtained from a tomographic reconstruction of the individual 2D projections measured at a time delay of 3.3 ps is shown in Supplementary Figure 2. The individual 2D projections were used in the masked-VMI analysis presented in Fig. 3 of the main article to determine the in-plane alignment distribution. Slices through the 3D ion momentum-distribution are also shown in Supplementary Figure 2. Tomographies were also acquired at a time delay of 3.3 ps for C^{2+} and $CH_xN^+ (x = 0, 1, 2)$ fragments. We note that minor orientation effects were observed due to the presence of the dc extraction field in the interaction region of the VMI spectrometer. For the $CH_xN^+ (x = 0, 1, 2)$ fragments, a degree of orientation $\langle \cos\theta_{2D} \rangle$ ranging from -0.05 to 0.05 was measured when the laser polarization was rotated by 90°. The 2D ion-momentum distributions of H^+ were thus symmetrized prior to the 3D tomographic reconstruction, such that the 3D momentum distributions in Supplementary Figure 2 represent the equal average over the four simultaneously present orientations, which are related via rotations of 180° around the *a* and *b* axes.

SUPPLEMENTARY NOTE 3: COMPUTATIONS

The rotational motion of indole was modeled in the rigid-rotor approximation using the rotational constants $A = 3877.9$ MHz, $B = 1636.1$ MHz, and $C = 1150.9$ MHz [1, 2]. The electric polarizability tensor for the equilibrium molecular geometry was computed *ab initio* at the CCSD/aug-cc-pVTZ [3, 4] level of theory in the frozen-core approximation. Electronic-structure calculations employed the quantum-chemistry package Dalton [5].

Time-dependent quantum dynamics simulations were performed using the general purpose code for quantum-mechanical modelling of molecule-field interactions RichMol [6]. In the simulations, the time-dependent wavefunction was built from a superposition of field-free eigenstates with time-dependent coefficients obtained by numerically solving the time-dependent Schrödinger equation. The latter was solved using the iterative approximation based on Krylov subspace methods, as implemented in the Expokit computational library [7]. The elliptically polarized alignment laser field was described as

$$E(t) = E_0(t) \left\{ \mathbf{e}_x \cos(\omega t)/\sqrt{3}, \mathbf{e}_z \sin(\omega t) \right\}, \qquad (2)$$

with $E_0(t)$, the electric field amplitude computed from the measured experimental peak intensity. The carrier frequency was fixed to $\omega = 2.354 \cdot 10^{15}$ Hz, corresponding to the central wavelength $\lambda = 800$ nm of the alignment

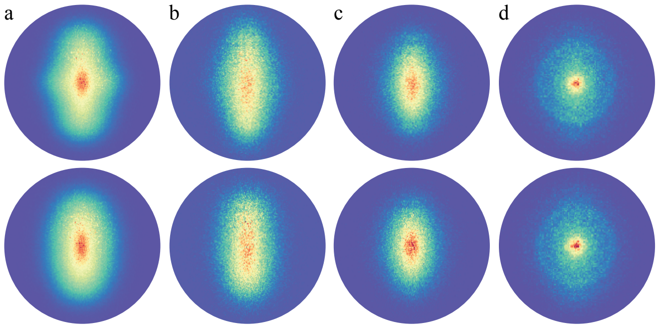

Supplementary Figure 1. 2D ion-momentum distributions of the fragments (a) C^+, (b) C_2^+, (c) $C_3H_x^+$, and (d) $C_4H_x^+$. The top row shows images obtained for the major alignment-laser-polarization axis vertical and parallel to the detector surface whereas the bottom row shows corresponding images with the minor alignment-laser-polarization axis vertical and parallel to the detector surface; cf. Fig. 1 in the main article.

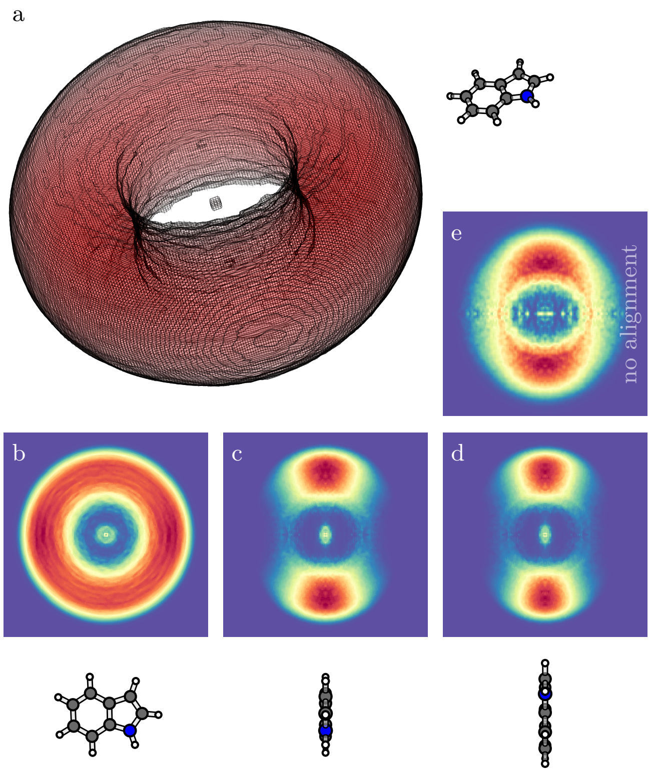

Supplementary Figure 2. Tomographically measured and reconstructed H^+ ion velocity distribution, displayed (a) as isosurface representation and (b–d) along slices through the center of the distribution. The ball-and-stick models of indole depict the relative orientations of the molecular fixed frame. Panel (e) shows a slice through the corresponding velocity distribution without laser alignment; its anisotropy results from the probe selectivity of the ionization laser.

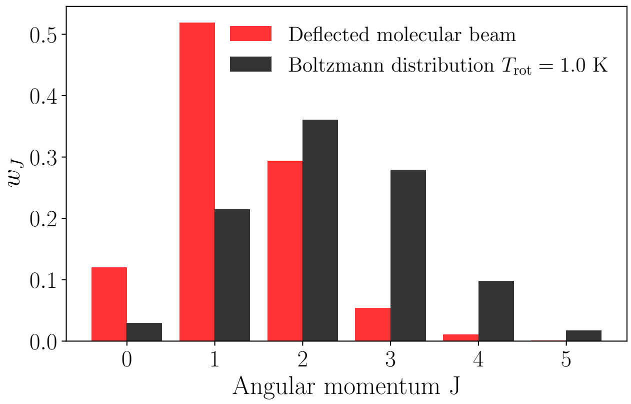

Supplementary Figure 3. **Comparison of the deflected-beam rotational-state distribution to a thermal Boltzmann distribution** with $T_{rot} = 1.0$ K in the undeflected molecular beam. The weights are the sum over all sublevels with the same angular momentum quantum number J. The two distributions are clearly different. In particular, the lowest energy rotational states have much higher weights in the deflected beam compared to a Boltzmann distribution.

laser. The time-dependent wavefunction was expressed in the basis of field-free rotational eigenstates of indole with all rotational states with $J \leq 30$ included and propagated on a time grid with a fixed time step of 10 fs. Convergence with respect to the size of the rotational basis set and the time step were carefully verified.

Since alignment depends nonlinearly on the laser intensity, which is not constant within the focal volume of the laser, integration of all simulated observables over the interaction volume is required. This has been approximated by repeating the calculations for five individual laser intensities, obtained by scaling the originally measured peak intensity I_0 with factors 0.2, 0.4, 0.6, 0.8, and 1.0. Focal volume averaging was carried out using the measured Gaussian beam profiles with widths (FWHM) of $\sigma_{align} = 56.4$ µm and $\sigma_{probe} = 28.2$ µm.

Finally, an incoherent average over the initial rotational-state distribution was carried out: The rotational-state distribution of the molecule behind the deflector was determined by fitting the measured vertical profile of the deflected molecular beam using `CMIfly` [8] and is depicted alongside a thermal distribution at 1 K in Supplementary Figure 3. We note that the inhomogeneous electrostatic field in the deflector leads to a spatial dispersion of rotational states according to their effective dipole moment, which is largest for the rotational ground state. By choosing an appropriate part in the molecular beam the contribution of the lowest-energy rotational states can be increased compared to a thermal Boltzmann distribution in the undeflected beam [9], as seen in Supplementary Figure 3.

In order to directly compare our simulations with the experiment and to characterize the degree of alignment, we computed the rotational probability-density distributions for all hydrogen and carbon atoms in the molecule. The evaluation of the rotational-density functions required the calculation of the Wigner rotation matrices, which was carried out using a Fourier-series based algorithm [10]. For different time delays, two-dimensional projections of the rotational probability density onto the YZ laboratory plane were computed for each atom individually, assuming axial recoil of the hydrogen ions along the C-H and N-H bond vectors, and for C^{2+} the vectors connecting the center of mass with each carbon atom were chosen as recoil axes. In analogy to the experiment, $\left\langle\cos^2\theta_{2D}\right\rangle^{sim}_{H^+,k}$ was extracted from these 2D projections for each hydrogen atom k. Furthermore, by rotating the simulated rotational probability-density around the laboratory Y-axis in steps of 1 ° and carrying out a 2D projection for each rotation angle, the tomography measurements were mimicked. As for the experiment, the signal within a radius of 20 pixels, calibrated to the experimental radius, was also integrated for each angle α_i and for every hydrogen and carbon atom to reproduce our masked-VMI measurements.

For hydrogens, the simulated time-dependent 2D alignment-revivals, $\left\langle\cos^2\theta_{2D}\right\rangle^{sim}_{H^+,k}$, and the angle-dependent integrated probability density, $D_{k,sim}(\alpha_i)$, at the center of the detector thus obtained were simultaneously fitted to the experiment by employing a least-squares fitting routine. The fitting was achieved by minimising the residual sum of squares, defined as:

$$\begin{aligned}\mathrm{RSS} = \sum_{i=1}^{N} \Bigg(&\left\langle\cos^2\theta_{2D}\right\rangle^{exp}_{H^+}(t_i) \\ &- \Big(\sum_{k=1}^{7} w_k \left\langle\cos^2\theta_{2D}\right\rangle^{sim}_{H^+,k}(t_i) + w_8\Big)\Bigg)^2 \\ &+ \left(\sum_{i=1}^{M}\Big(D_{exp}(\alpha_i) - \sum_{k=1}^{7} w_k G(\alpha) * D_{k,sim}(\alpha_i)\Big)\right)^2 ,\end{aligned} \tag{3}$$

with the total number $N = 405$ of time steps t_i and the total number $M = 90$ of measured angles α_i in the angle-dependent integrated probability density. w_k and $k = 1-7$ were weighting factors associated to each hydrogen atom k and w_8 was an offset that accounted for the geometric alignment. $G(\alpha) \propto \exp(-\alpha^2/2w_9^2)$ was a Gaussian function with opening angle w_9 (standard deviation) used to account for the non-axial recoil of the hydrogen atoms. In the fitting procedure, a total of nine fitting parameters, w_k, $k = 1 \ldots 9$, were used for a total of 495 measured data points. Best agreement was achieved for $\mathrm{RSS}_{min} \approx 0.06$ for the fitting parameters shown in Supplementary Table I. The resulting fits are shown in Fig. 2 b and Fig. 3 in the main article, respectively. Comparison of our measured revival dynamics with the theoretical fit results in a normalized χ^2 value of 2.3, meaning, on

Supplementary Table I. **Parameters** obtained from the fit of the H^+ and C^{2+} measurements shown in the main manuscript using the model outlined in the text.

Weights	H^+	C^{2+}
$w_1 + w_3$	0.07 ± 0.16	
w_2	0.21 ± 0.06	0.07 ± 0.01
w_3		0.34 ± 0.01
$w_4 + w_7$	0.42 ± 0.11	0.06 ± 0.02
$w_5 + w_6$	0.30 ± 0.14	0.12 ± 0.03
$w_{3a} + w_{7a}$		0.41 ± 0.02
w_8	0.081 ± 0.021	
w_9	50°	

average, our model deviates from the measured values by approximately 1.5 times the standard error of the measured values. A similar procedure was used to fit the angle-dependent integrated probability density measured in the C^{2+} ions. In this case, eight weighting factors, corresponding to the eight carbon atoms of indole, were used as fitting parameters. We note that for the fit of the C^{2+} ions the Gaussian function accounting for non-axial recoil was not necessary to achieve a very good fit to the experimental data. The parameters retrieved from the fit are also given in Supplementary Table I.

Due to averaging of the four orientations of indole the least squares fitting procedure for hydrogen and carbon atoms with similar angles with respect to the z_I axis of indole, e. g., H_4 & H_7, see Fig. 1 a in the main article, resulted in revivals and angle-dependent probability distributions that are indistinguishable from each other in the experiment. This was further confirmed by computing the covariance matrix, showing very strong correlations for near similar hydrogen and carbon atoms, whereas no significant correlations were found between the others. The total probabilities for these atoms were thus summed.

The expectation values of the alignment cosines were computed for the three main polarizability axes in the principle-axis polarizability frame with respect to the laboratory-fixed frame by employing Monte-Carlo integration with a convergence better than 10^{-3} using $\sim 10^5$ sampling points. The simulated degree of alignment for the main polarizability axes of the molecule $\alpha_{z_I} > \alpha_{x_I} > \alpha_{y_I}$ with respect to the laboratory axes XYZ is shown in Supplementary Figure 4. The highest achieved 3D degree of alignment was thus characterized to be $\left\langle\cos^2\theta_{Zz_I}\right\rangle = 0.88$, $\left\langle\cos^2\theta_{Yy_I}\right\rangle = 0.83$, $\left\langle\cos^2\theta_{Xx_I}\right\rangle = 0.85$, and $\left\langle\cos^2\delta\right\rangle = 0.89$, where $\cos^2\delta = \frac{1}{4}(1 + \cos^2\theta_{Zz_I} + \cos^2\theta_{Yy_I} + \cos^2\theta_{Xx_I})$ [11].

As stated in the main article, the laser field drops to below 1 % of the peak intensity within 3.3 ps after its peak value, i. e., to within the noise level of the measurement. The degree of field-free alignment also assumes its maximum value at 3.3 ps, which we defined as the start

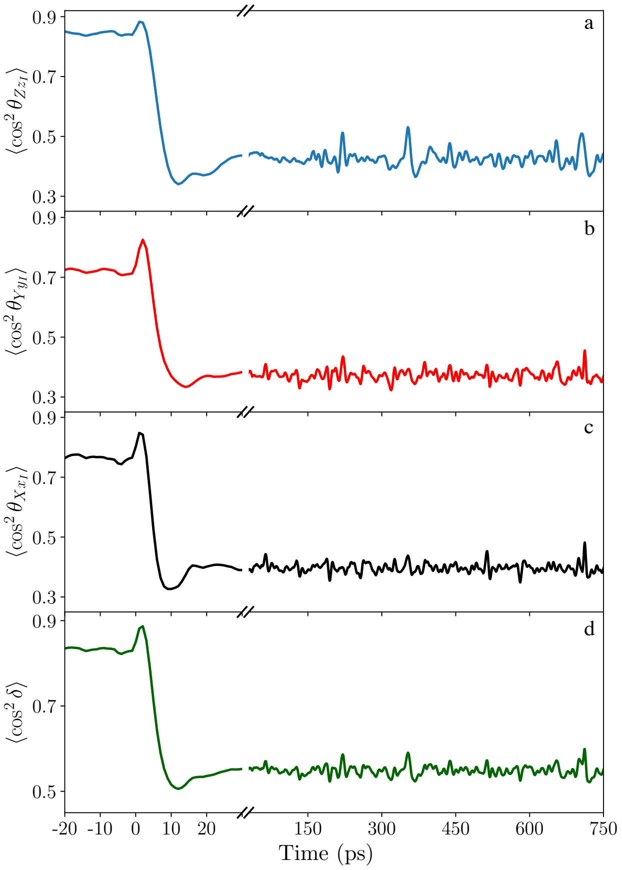

Supplementary Figure 4. **Simulated degree of 3D alignment** characterized through the expectation values (a) $\left\langle\cos^2\theta_{Zz_I}\right\rangle$, (b) $\left\langle\cos^2\theta_{Yy_I}\right\rangle$, (c) $\left\langle\cos^2\theta_{Xx_I}\right\rangle$ and (d) $\left\langle\cos^2\delta\right\rangle$ with the cartesian principal axes of the polarizability tensor frame z_I, y_I, x_I, the cartesian axes of the laboratory-fixed frame X, Y, Z, and (d) $\cos^2\delta$ [11]; see text for details.

of the field-free region. Simulations have been carried out considering the effect of a residual laser field on the order of 0.1% and 1% of the peak intensity of the alignment laser field and compared to the completely field-free case, shown in Supplementary Figure 5. In the simulations, the field was set to either 0.1% or 1% at $t = 3.3$ ps. The degree of alignment in the region of interest, until 13 ps where an, generally unwanted, postpulse appears, does not show any differences, even with such a small residual field present.

Calculations of the expected alignment pulse shape, taking into account SLM pixelation, SLM pixel gaps, the laser beam diameter, and the spectral spread at the Fourier plane, resulted in an expected laser intensity between $t = 4$ ps and $t = 10$ ps to be a factor of 70 lower than at the peak of the alignment pulse at $t = 0$ ps.

Finally, we note that further calculations for indole (not shown) indicate that a truncation time of ≤ 2 ps is required to obtain essentially identical dynamics to having an instantaneous truncation. Therefore, phase shaping

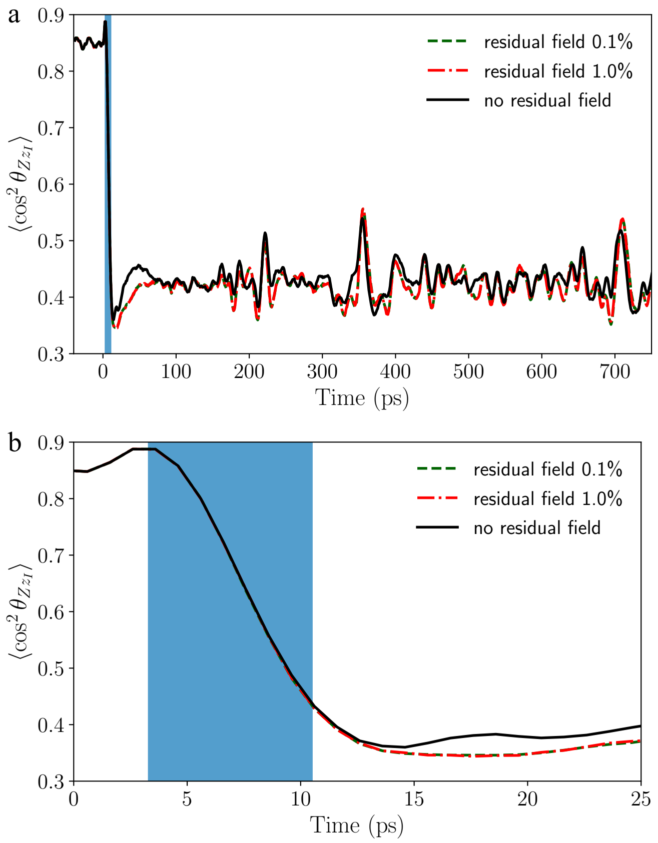

Supplementary Figure 5. **Effect of residual laser fields on the alignment.** Comparison of the 3D degree of alignment of the main polarizability axis z_I with respect to the laboratory Z axis $\langle \cos^2 \theta_{Zz_I} \rangle$ without any residual field, with a residual field of 0.1% of the peak intensity and with a residual field of 1% of the peak intensity. (a) Full range with revivals, (b) zoom into the early times after the peak of the alignment laser field. In both panels the field-free region of interest is marked in blue.

using the SLM based shaper was highly advantageous, or simply necessary, instead of the more simple frequency filter used in [12], which would result in a 8 ps fall-off in the best case.

SUPPLEMENTARY REFERENCES

[1] G. Berden, W. L. Meerts, and E. Jalviste, Rotationally resolved ultraviolet spectroscopy of indole, indazole, and benzimidazole: Inertial axis reorientation in the $S_1(^1L_b) \leftarrow S_0$ transitions, J. Chem. Phys. **103**, 9596 (1995).

[2] C. Kang, T. M. Korter, and D. W. Pratt, Experimental measurement of the induced dipole moment of an isolated molecule in its ground and electronically excited states: Indole and indole–H_2O, J. Chem. Phys. **122**, 174301 (2005).

[3] T. H. Dunning, Gaussian basis sets for use in correlated molecular calculations. I. The atoms boron through neon and hydrogen, J. Chem. Phys. **90**, 1007 (1989).

[4] R. A. Kendall, T. H. Dunning, Jr., and R. J. Harrison, Electron affinities of the first-row atoms revisited. Systematic basis sets and wave functions, J. Chem. Phys. **96**, 6796 (1992).

[5] K. Aidas, C. Angeli, K. L. Bak, V. Bakken, R. Bast, L. Boman, O. Christiansen, R. Cimiraglia, S. Coriani, P. Dahle, E. K. Dalskov, U. Ekström, T. Enevoldsen, J. J. Eriksen, P. Ettenhuber, B. Fernández, L. Ferrighi, H. Fliegl, L. Frediani, K. Hald, A. Halkier, C. Hättig, H. Heiberg, T. Helgaker, A. C. Hennum, H. Hettema, E. Hjertenæs, S. Høst, I.-M. Høyvik, M. F. Iozzi, B. Jansík, H. J. Aa. Jensen, D. Jonsson, P. Jørgensen, J. Kauczor, S. Kirpekar, T. Kjærgaard, W. Klopper, S. Knecht, R. Kobayashi, H. Koch, J. Kongsted, A. Krapp, K. Kristensen, A. Ligabue, O. B. Lutnæs, J. I. Melo, K. V. Mikkelsen, R. H. Myhre, C. Neiss, C. B. Nielsen, P. Norman, J. Olsen, J. M. H. Olsen, A. Osted, M. J. Packer, F. Pawlowski, T. B. Pedersen, P. F. Provasi, S. Reine, Z. Rinkevicius, T. A. Ruden, K. Ruud, V. V. Rybkin, P. Sałek, C. C. M. Samson, A. S. de Merás, T. Saue, S. P. A. Sauer, B. Schimmelpfennig, K. Sneskov, A. H. Steindal, K. O. Sylvester-Hvid, P. R. Taylor, A. M. Teale, E. I. Tellgren, D. P. Tew, A. J. Thorvaldsen, L. Thøgersen, O. Vahtras, M. A. Watson, D. J. D. Wilson, M. Ziolkowski, and H. Ågren, The Dalton quantum chemistry program system, WIREs Comput. Mol. Sci. **4**, 269 (2014).

[6] A. Owens and A. Yachmenev, RichMol: A general variational approach for rovibrational molecular dynamics in external electric fields, J. Chem. Phys. **148**, 124102 (2018), arXiv:1802.07603 [physics].

[7] R. B. Sidje, Expokit: a software package for computing matrix exponentials, ACM Trans. Math. Soft. **24**, 130 (1998).

[8] Y.-P. Chang, D. Horke, S. Trippel, and J. Küpper, CMI-fly, `https://github.com/CFEL-CMI/cmifly` (2020), originally published in [9].

[9] Y.-P. Chang, D. A. Horke, S. Trippel, and J. Küpper, Spatially-controlled complex molecules and their applications, Int. Rev. Phys. Chem. **34**, 557 (2015), arXiv:1505.05632 [physics].

[10] N. Tajima, Analytical formula for numerical evaluations of the Wigner rotation matrices at high spins, Phys. Rev. C **91**, 014320 (2015).

[11] V. Makhija, X. Ren, and V. Kumarappan, Metric for three-dimensional alignment of molecules, Phys. Rev. A **85**, 033425 (2012).

[12] A. Chatterley, E. T. Karamatskos, C. Schouder, L. Christiansen, A. V. Jörgensen, T. Mullins, J. Küpper, and H. Stapelfeldt, Switched wave packets with spectrally truncated chirped pulses, J. Chem. Phys. **148**, 221105 (2018), arXiv:1803.03953 [physics].
